# Career and life planning in the context of the postgraduate medical training – current challenges and opportunities

**DOI:** 10.3205/zma001660

**Published:** 2024-02-15

**Authors:** Stella Oberberg, Elena K. Enax-Krumova, Christiane Kruppa, Christine H. Meyer-Frießem, Robin Denz, Alina Funhoff, Vanessa Behrens, Dinah Berres, Vera Fortmeier, Dina Sträter, Johanna Strotmann, Maxi von Glinski

**Affiliations:** 1 St Josef Hospital, Clinic for Orthopaedics and Trauma Surgery, Bochum, Germany; 2 BG University Hospital Bergmannsheil gGmbH, Department of Neurology, Bochum, Germany; 3 BG University Hospital Bergmannsheil gGmbH, Department of General and Trauma Surgery, Bochum, Germany; 4 BG University Hospital Bergmannsheil gGmbH, Department of Anaesthesiology, Intensive Care and Pain Medicine, Bochum, Germany; 5 Klinikum Lünen, KLW St. Paulus GmbH, Department of Anaesthesiology, Intensive Care and Pain Medicine, Lünen, Germany; 6 Ruhr University Bochum, Department of Medical Informatics, Biometry and Epidemiology, Bochum, Germany; 7 University Hospital Knappschaftskrankenhaus Bochum GmbH, Department of Neurology, Bochum, Germany; 8 BG University Hospital Bergmannsheil gGmbH, Clinic for Cardiac and Thoracic Surgery, Bochum, Germany; 9 Heart and Diabetes Centre NRW, Bad Oeynhausen, Germany; 10 St Josef Hospital, Department of General and Visceral Surgery, Bochum, Germany; 11 BG University Hospital Bergmannsheil gGmbH, Department of Plastic and Hand Surgery, Burn Center, Bochum, Germany

**Keywords:** postgrostgraduate medical training, career, gender, female physician, male physician, parental leave, part-time work, work schedules

## Abstract

**Introduction:**

The possibility of balancing career and family is meanwhile a central concern for most physicians when choosing a job. The aim of this study was to identify current barriers and opportunities for physician education and career planning.

**Methods:**

This cross-sectional study was conducted as an online survey between 11/2021 and 02/2022 and targeted physicians at all career levels in Germany who were members of a clinical professional association. Alternative and consent questions were used to assess experiences/attitudes toward various aspects of life and career planning, as well as alternative work and parental leave models, depending on gender, specialty, and hierarchical level.

**Results:**

The majority of the 2060 participants were female (69%) and had children (66%). Many childless residents reported that they felt they had to choose between children and a career. The majority of female residents, specialists and attending physicians (Ø 55.5%) stated that they had experienced career losses as a result of taking parental leave, while most men did not share this experience (Ø 53.7%). 92% of all participants agreed with the statement that men and women have different career opportunities. Job-sharing models were considered feasible at all levels of the hierarchy by an average of 55.6% of all medical executives.

**Conclusion:**

Parenthood and the use of parental leave and part-time work appear to have a significant impact on the career paths of those surveyed. Although the majority of directors of medical training programs are open to job-sharing models, further measures are needed in order to equalize career opportunities for men and women.

## Zusammenfassung

**Einführung:**

Die Möglichkeit der Vereinbarkeit von Karriere und Familie ist für die meisten Ärzt*innen mittlerweile ein zentrales Anliegen bei der Stellenauswahl. Ziel dieser Studie war es, aktuelle Hindernisse und Chancen für die Weiterbildungs- und Karriereplanung von Ärzt*innen zu identifizieren.

**Methoden:**

Die Querschnittsstudie erfolgte als Onlinebefragung im Zeitraum 11/2021-02/2022 und richtete sich an Ärzt*innen aller Karrierestufen in Deutschland, die einer klinischen Fachgesellschaft zugehörig waren. Mittels Alternativ- und Zustimmungsfragen wurden die Erfahrungen/Einstellungen zu verschiedenen Bereichen der Lebens- und Karriereplanung sowie alternativer Arbeits- und Elternzeitmodelle in Abhängigkeit von Geschlecht, Fachdisziplin und Hierarchieebene erfragt.

**Ergebnisse:**

Der Großteil der 2060 Teilnehmer*innen war weiblich (69%) und hatte Kinder (66%). Viele kinderlose Assistenz-/Fachärzt*innen gaben an, das Gefühl zu haben, sich zwischen Kind und Karriere entscheiden zu müssen. Ein Großteil der Assistenz-, Fach- und Oberärztinnen (Ø 55,5%) war der Meinung, durch die genommene Elternzeit Karriereeinbußen erlebt zu haben, während die meisten Männer diese Erfahrung nicht teilten (Ø 53,7%). 92% aller Teilnehmer*innen stimmten mit der Aussage überein, dass sich die Karrierechancen von Männern und Frauen unterscheiden. Job-Sharing-Modelle wurden von durchschnittlich 55,6% aller ärztlichen Führungskräfte für umsetzbar auf allen Hierarchieebenen gehalten.

**Schlussfolgerung:**

Elternschaft und die Inanspruchnahme von Eltern- und Teilzeit scheinen bei den Befragten einen wesentlichen Einfluss auf den Karriereverlauf zu haben. Auch wenn der Großteil der weiterbildenden Führungskräfte Job-Sharing-Modellen offen gegenübersteht, gilt es weitere Maßnahmen zu ergreifen, um die Karrierechancen von Männern und Frauen anzugleichen.

## Introduction

The possibility of a family- and life-friendly career is now seen by many physicians as a necessary prerequisite for a (postgraduate medical training) position [1], [2], [3]. Over the last two decades, a wide range of family policy reforms have created the theoretical basis for both women and men to take time off work or reduce their working hours. This allows them to spend more time with their children and facilitates childcare [4], [5]. Despite this, women in particular still perceive the compatibility of family and a medical career as inadequate [6].

Two-thirds of all medical students and employees in healthcare are female [7], [8]. However, the proportion of female clinic directors at university hospitals remained unchanged at just 13% in 2022, while the proportion of female senior physicians had risen to 37% compared to 31% in the previous survey in 2019 [9].

At the same time, two-thirds of working mothers work part-time, which is often associated with fewer career opportunities, compared to 7.6% of working fathers who work part-time [10]. 

To ensure that existing family policies lead to a long-term equalization of career opportunities and do not exacerbate existing disadvantages, it is essential that they are supported and advocated, especially by directors of postgraduate medical training programs [3]. To date, the literature has largely reflected the perspectives of medical residents on existing working conditions [6], [8], [11]. However, the opinions of senior and chief physicians regarding gender-specific career opportunities and alternative working time models appear to be underrepresented. 

The aim of this study was to investigate differences in family and life planning among physicians during and after training, as well as their attitudes towards potential improvement strategies, according to gender, specialty (surgical/conservative), and hierarchical level.

## Methods

After obtaining positive ethics approval (Faculty of Medicine, Ruhr University Bochum: 21-7298), data collection of this cross-sectional study took place between 11/2021 and 02/2022. Physicians of all specialties, career levels, and age groups (>18 years) who voluntarily participated in the German online survey were included. For this purpose, an electronic letter with a link to an anonymous, self-administered questionnaire was sent to the offices of 145 clinically relevant professional associations and registered medical societies in Germany and asked to be forwarded via their mailing lists. The questionnaire itself was hosted on the online survey platform LimeSurvey. The time required to complete the survey was estimated at approximately four to a maximum of thirteen minutes. 

Written informed consent about the anonymity of the survey and consent for active and voluntary participation was obtained before the survey began. Sociodemographic data were collected at the beginning of the survey: Age group, gender, physician position (resident, specialist, senior, chief), type of clinic/department, marital status, number of children living in the household. Based on this information, participants were directed to alternative pathways with adapted questions regarding part-time/full-time employment and reasons for this, desire or plan to have children if childless, whether/how long parental leave was taken (1-2/3-6/7-12/>12 months), expected/actual career losses due to parental leave, attitudes towards career opportunities for women and men, division of domestic duties, attitudes towards job sharing models, etc. In addition to their own life and career planning, physicians in leadership positions (senior and chief physicians) were asked about their attitudes toward part-time work and parental leave for employees and whether this is supported. 

Both alternative (yes/no) and agreement questions using a 5-point Likert scale (1=does not apply, 2=does rather not applyl, 3=does somewhat apply, 4=more likely to apply, 5=applies completely) were used.

### Statistics

Due to the exploratory nature of the data collection, descriptive analysis was used to compare in particular the opinions/life and career plans of women and men, surgical and conservative physicians as well as physicians in different hierarchical positions. Accordingly, no p-values are being reported [12].

The graphs were analyzed and generated using the R programming language, version 3.2.1. The numerical variables are displayed in percentages.

## Results

A total of 2060 questionnaires were fully (N=1665) or partially (N=395) completed and thus included in the analysis. 

Table 1 (Tab. 1) summarizes the sociodemographic characteristics of the participants. The majority were female (69.4%) and between 31 and 45 years old (51.6%). 23.4% of the physicians were undergoing postgraduate medical training. The majority of physicians worked as specialists or senior physicians (32.7% and 32.3%, respectively). 88.5% of all participants were in a relationship, 74.4% had children.

### Children & career

When differentiating the responses by position and gender, the majority of participants at specialist level and above reported having children, see attachment 1 . 27.6% of the female senior physicians were childless, compared with 19.8% of male senior physicians, and 44.6% of female chief physicians were childless, compared with 23.9% of their male colleagues. 

The majority of childless residents and specialists reported that they (rather) felt they had to choose between children and their career, with the proportion of women agreeing with this statement being significantly higher (residents: women 64.4% vs. men 48.4%; specialists: women 69.9% vs. men 45.5%). The majority of senior physicians (43.9%) and chief physicians (47%) still (tended to) agree with this statement. The majority of residents and specialists indicated that they (tend to) consider career advancement when planning to have children. In addition, 61.8% of female senior physicians and 43.5% of male senior physicians (rather) agreed with this statement.

### Parental leave

The majority of both female (76.9%) and male physicians in postgraduate medical training (90.3%) with a child/children stated that they had already taken parental leave during their training. The majority of respondents without children could also imagine taking parental leave during (male: 59.3% vs. female: 55.2%) or after their training (male: 37% vs. female: 42.2%).

87.8% of the female specialists and 75,4% of male specialists had already taken parental leave. In contrast, only 31.3% of male and 42.2% of female senior physicians reported that they have taken parental leave. At the level of chief physicians, 20% of the men and 64% of the women had taken parental leave. 

**Figure 1:**
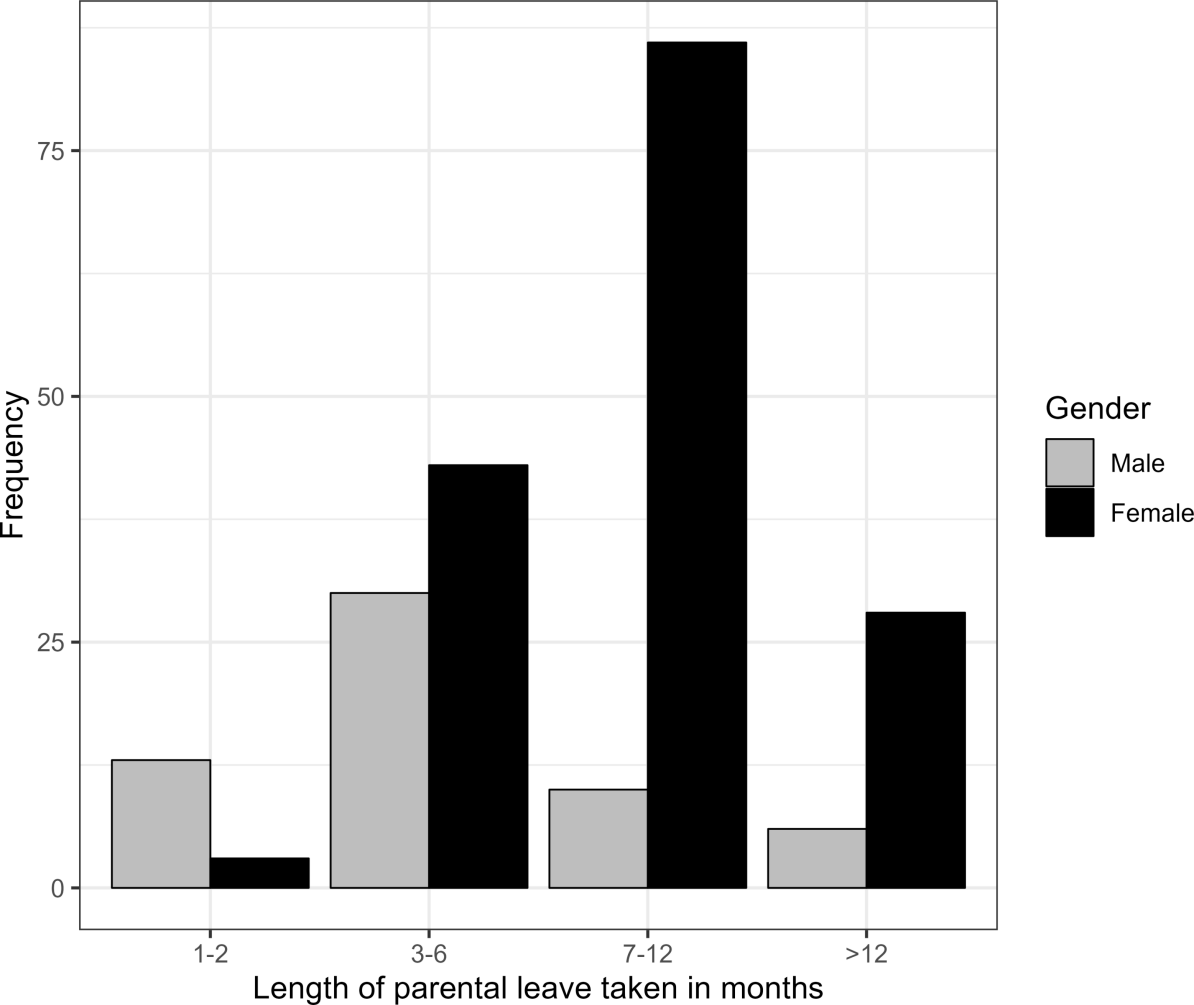
Length of parental leave taken in months depending onthe gender

The length of parental leave taken was in 36,7% in the group of male residents 1-2 months, in 46,7% 3-6 months, while 91,7% of the female residents took at least 7 months. A similar discrepancy was found among specialists (73.4% of men took parental leave for 1-2 and 3-6 months equally; 89.7% of women for at least 7 months) and senior physicians (men: 68% for 1-2 and 3-6 months equally; women: 71.9% for at least 7 months), see figure 1 (Fig. 1).

Among childless residents/specialists, the majority (tended to) agree/agreed with the statement that parental leave worsens career opportunities in the long term (residents: 64.1% male, 77.1% female; specialists: 68.4% male, 88.4% female). Among senior physicians, 34.2% of men and 60.3% of women shared this opinion. 

Among female residents and female specialists who had already taken parental leave, the majority (67.1% and 62.2%, respectively) believed that they had (rather) experienced career losses as a result. Among senior physicians, 48.4% (tended to agree) agreed with this statement. In contrast, the majority of residents (41.4%), specialists (60.0%) and senior consultants (58.7%) did not (tend to) share this opinion.

### Part time employment

The majority of respondents were employed full-time. By position, 31.5% of female residents were employed part-time (compared with 7.5% of the males). The difference in part-time employment was even greater between female (53.5%) and male residents (14.6%). 41.9% of the female senior physicians were employed part-time (compared with 13.8% of the males). 46.7% of female chief physicians reported having worked part-time during their career (compared with 17.1% of the males). For both genders, the most common reason for part-time employment was child care (men: 66.7%; women: 90.0%).

### Domestic and family obligations

50% of male residents reported that domestic/family responsibilities were shared equally, with another 48.9% reporting that their female partner was primarily responsible. Correspondingly, 36.9% of female residents were primarily responsible for these duties, compared with 50.7% of female specialists and 35.5% of female senior physicians. Among male senior physicians, 74.7% reported that their female partner was primarily responsible for and family duties, compared with only 18% of female chief physicians reporting to be supported by their partners.

### Career opportunities and future vision

**Figure 2:**
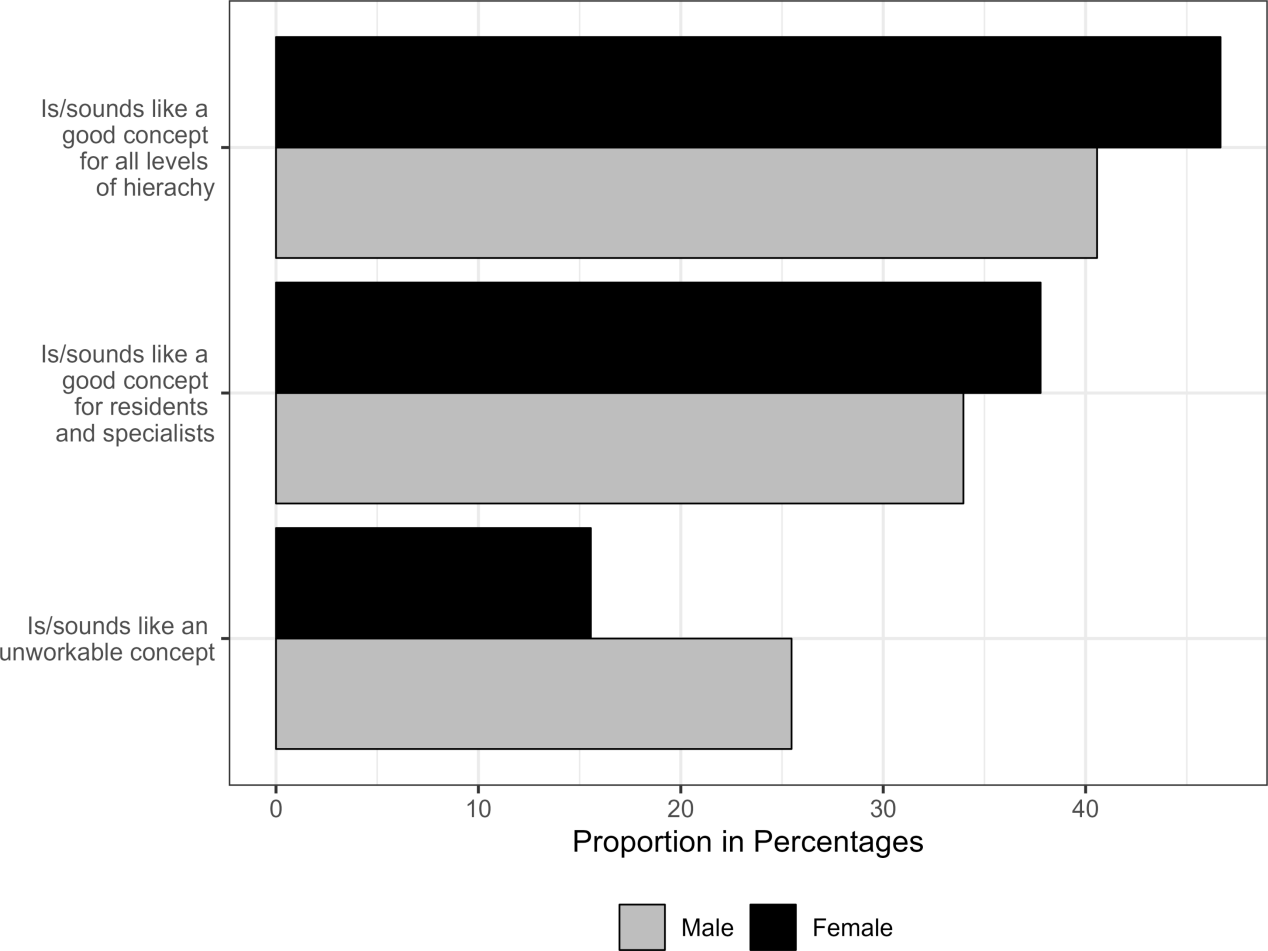
Chief physicians’ responses to the question: What do you think about job sharing models (e.g., 2 people sharing a 100% position)?

Attachment 2 summarizes all questions and answers on balancing between work and family. 91.9% of all participants (tended to agree) agreed with the statement that men and women have different career opportunities. Regardless of their position, female physicians agreed significantly more often with this statement (ranging from 95.4% of female senior physicians to 97.8% of female residents) than male physicians at all career levels (ranging from 78.3% of female senior physicians to 80.2% of male residents). 70.1% of all respondents also (rather) stated that the introduction of the "Scandinavian model", meaning an equalization of the length of parental leave for men and women, would lead to an equalization of career opportunities for men and women, with no significant discrepancy between positions and genders. While the majority of senior physicians (m: 54.1%; f: 62.3%) considered job-sharing models to be a good concept at all hierarchical levels, 43.4% of chief physicians considered the concept to be applicable to all hierarchical levels. Further 35.7% of chief physicians could imagine job sharing at least at the resident and specialist levels, see figure 2 (Fig. 2).

### Differences depending on the speciality

When comparing work in surgical and conservative specialties, a significantly higher proportion of physicians working in surgery (tended to agree) agreed with the statement that they had the impression to need to choose between career and children (76.3% vs. 48.7%) and that they orient themselves according to their career steps when planning to have children (78.6% vs. 57.5%), see attachment 3 . 60% of the physicians in surgical disciplines who wanted to have children planned to take parental leave not until completing their training (vs. 37.3% of conservative physicians). A slightly higher proportion of participants with surgical disciplines also believed that parental leave would (rather) reduce their chances of career advancement in the long term (67% vs. 52.5%). After taking parental leave, the proportion of physicians in both groups who felt that their career opportunities were (rather) impaired was almost similar (54.3% operative and 52.5% conservative). A slightly higher proportion of conservative physicians in leading positions (senior physicians and chief physicians) reported that they supported employees to take parental leave (conservative: 92.4% vs. operative: 85.9%) or working part-time (conservative: 86.7% vs. operative: 79.3%).

## Discussion

The purpose of this study was to identify current barriers and opportunities in physicians’ postgraduate medical training and career planning related to work-life balance. To our knowledge, this is the first study to include senior and chief physicians in the survey. This made it possible to compare opinions depending on career position not only reflecting the wishes, concerns and views of trainees, but also highlights possible ways in which these can be better addressed in the future. 

In summary, our study shows that the majority of physicians in Germany who are in a partnership already have children and, if not, would like to have children. At the same time, concerns about career losses because of taking parental leave are common. Many participants, especially women, align their family plans towards their career steps or even feel that they must choose between children and career. Job-sharing models and the possibility of more "partner months" corresponding to the Scandinavian model were perceived by most participants as opportunities to further improve and equalize career opportunities in a life- and family-friendly context.

In line with the current literature, our findings also show that still mainly women take parental leave, work part-time subsequently, and are primarily responsible for domestic tasks. Despite the high proportion of women in medicine, this is still associated with considerable career losses for female physicians [8], [9], [13], [14], [15], [16], [17].

One-third of the female chief physicians surveyed indicated that they had interrupted their careers for parental leave, and two-thirds of them for as long as 7-12 months. This indicates that it is possible for women in Germany to take parental leave and still hold a leadership position. At the same time, almost half of the female chief physicians in our cohort are childless, compared with only one-fifth of childless male chief physicians. This corresponds to the findings of a US study stating that female plastic surgeons had children significantly less often and much later than their male colleagues in the same position [18]. In our cohort, which cannot be considered as representative of all German physicians, the same phenomenon is evident starting at senior physician level. One possible explanation lies in the alternative life planning required by the intensive work commitment of a leading physician. As the question on the division of domestic responsibilities shows, this work commitment is more likely to be covered by the partners of male colleagues in their private life. In addition, structures such as corporate childcare facilities with compatible opening hours are very important in minimizing the need to choose between family and work [3], [16].

In our survey, 62% of respondents with children reported having taken parental leave. The proportion of men who had taken parental leave was as high as almost 40% at the resident, specialist and senior consultant level. Half of the male physicians surveyed did not see any long-term limitations of career opportunities as a result of taking parental leave. On the one hand, this may be due to the fact that their careers were already well advanced prior taking parental leave resulting in fewer disadvantages, or it may be due to subjective attitudes towards their careers. On the other hand, it may also indicate an increasing acceptance and support of parental leave by superiors. The fact that a high percentage of women at all hierarchical levels perceived disadvantages as a result of taking parental leave may be due to the duration of parental leave. While the majority of women took at least 7 months of parental leave, most men took a maximum of 6 months.

Our survey found that more female physicians than male physicians continue to work part-time, mostly due to child care. Similarly, an earlier study on family planning found that 76% of female physicians wanted to work part-time, while only 16% of male physicians expressed that wish [8]. Similarly, in this survey, the proportion of women working part-time as a result of having children increased significantly more (from 4% to 50%) than that of men (from 2% to 15%) [6]. 64% of female physicians working part-time also reported that they felt disadvantaged in their postgraduate medical training because of their part-time work [6]. 

More than three-quarters of male respondents and almost all female respondents agreed that career opportunities are different for men and women. 

Female doctors tend to be less advanced in their careers and choose less prestigious medical specialties and career paths [8], [11], [14]. The reasons for the persistent "gender bias" may lie in the multifactorial burden on working women due to childcare and primary responsibility for domestic tasks, or in the longer periods of work absence due to parental leave followed by part-time work. Part-time employment itself leads to a significant extension of the period of specialist training, if not already completed. To equalize the career opportunities of women and men, working environment and perhaps even individual life planning needs to be rethought. A study by Raspe et al. found that the more ambitious the hospital career the shorter was the parental leave taken [6]. Theoretically, it is already possible to divide the duration of parental leave equally between both parents. In practice, however, this does not seem to be possible or desirable in most partnerships. The introduction or facilitation of more “partner months” could be understood as a possible politically driven incentive here.

In addition, mentoring programs at an early stage of training can support physicians in their individual career and life planning [19].

Job-sharing models received a high level of approval in our survey. This would also enable part-time employees to occupy leadership positions. And even during medical specialist training, they can lead to more effective “deployment” of part-time employees and thus improve staff coordination for employers. Although, at first glance, the results of our study confirm the already known career differences between women and men, with a predominantly traditional division of family roles, they offer an optimistic view of the future. They show that there is a high level of awareness of the continuing inequality of opportunities between women and men. And they show that the majority of leading physicians are not fundamentally opposed to alternative work models. Based on the results of these exploratory studies, further research can be planned in which a quantitative, statistically meaningful analysis can be carried out by defining appropriate hypotheses in advance.

## Limitations

The main limitation of this study is the cross-sectional design with incomplete representativeness based on a heterogeneous collective of professional associations with a majority of female participants. It is also obvious that there is a high participation of female physicians who have already dealt with the issues investigated. Finally, selection bias cannot be ruled out.

## Conclusion

Almost all physicians perceive the career opportunities of women and men to be unequal, both during and after training. Gender mainstreaming therefore remains a key issue. Many physicians, especially women, align their family planning according to career steps, which suggests impaired career opportunities due to parental leave during specialist training, or even have the impression that they have to choose between children and a career. This can only be addressed by innovative work and parental leave models, in addition to the much-discussed requirements for reconciling career and family, in particular the expansion of corporate childcare.

We have been able to show that besides the women the majority of mentake parental leave. In contrast, they do not fear or perceive any long-term effects on career opportunities. At the same time, many leading physicians and therefore coordinators of postgraduate medical training, seem to support men and women equally in terms of parental leave and part-time work. This suggests that the work environment is already undergoing significant changes. Further research and interventions are needed to provide a sufficient platform for this issue.

## Authors’ ORCIDs

- Stella Oberberg: 0000-0002-3761-1986
- Elena K. Enax-Krumova: 0000-0002-6162-9414
- Christiane Kruppa: 0000-0001-6817-9949
- Christine H. Meyer-Frießem: 0000-0001-8743-2065
- Robin Denz: 0000-0002-2682-5268
- Alina Funhoff: 0009-0007-7802-9742
- Vanessa Behrens: 0009-0007-0818-7235
- Dinah Berres: 0009-0008-2087-6348
- Vera Fortmeier: 0000-0001-5852-5617
- Dina Sträter: 0009-0002-9029-6070
- Johanna Strotmann: 0000-0001-7847-6138
- Maxi von Glinski: 0000-0002-3923-896X

## Acknowledgements

The project was implemented as part of the mentoring programme “MentÄ- Successfull in clinic, science and teaching” at Ruhr-Universität Bochum (2020-2022), which was funded by the Lore Agnes Foundation and the Faculty of Medicine [19]. 

EEK holds an endowed professorship funded by the German Social Accident Insurance (DGUV) for a period of 6 years (2020-2026).

## Competing interests

The authors declare that they have no competing interests. 

## Supplementary Material

Personal life planning depending on position and gender

Opinion on work-life balance depending on position and gender

Views on work-life balance depending on whether you work in a surgical or conservative specialty

## Figures and Tables

**Table 1:**
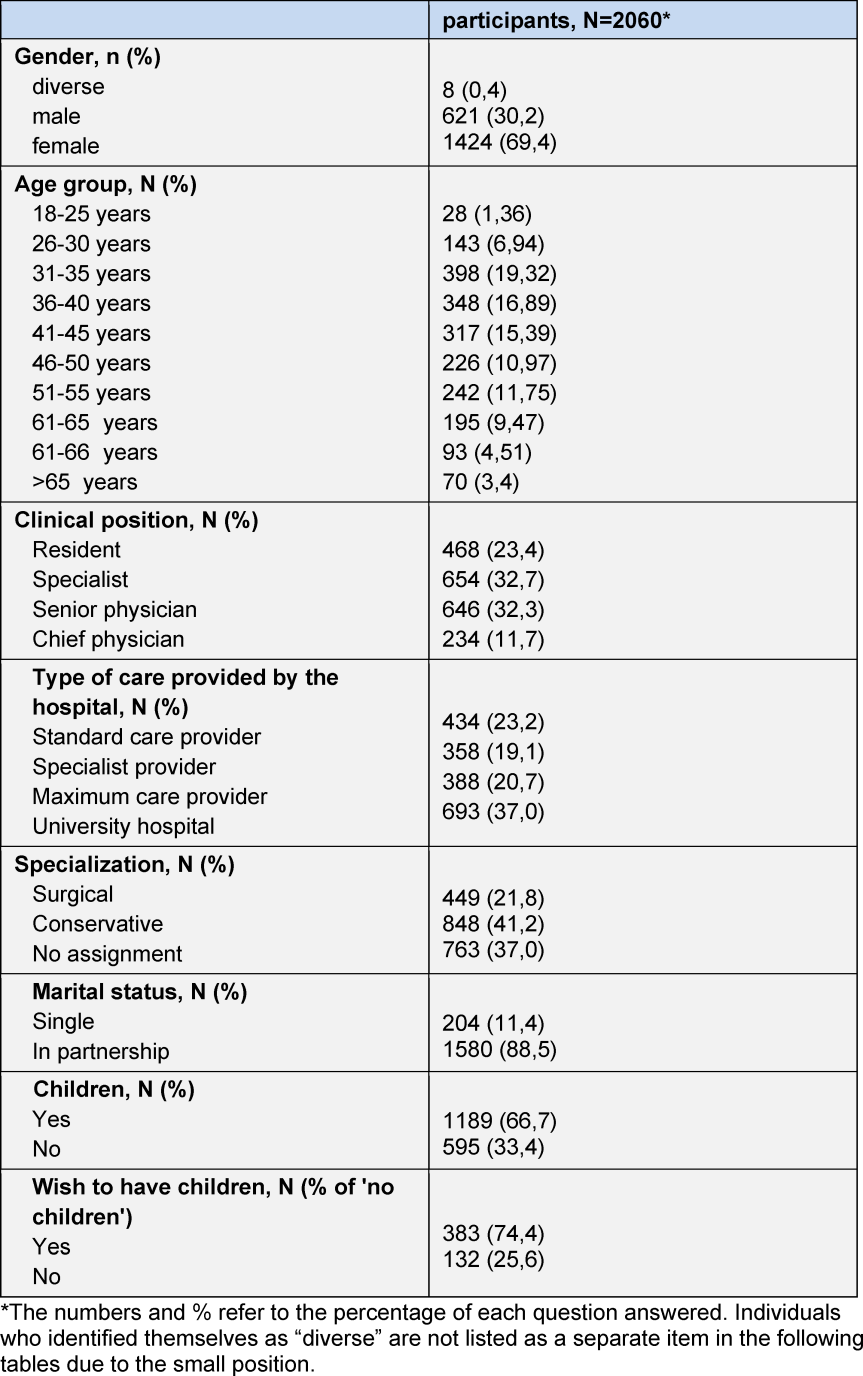
Socio-demographic characteristics of the participant collective
