# Supplementary material for: Career and life planning in the context of the postgraduate medical training – current challenges and opportunities
Source: GMS J Med Educ. 2024 Feb 15;41(1):Doc5. doi: 10.3205/zma001660 (PMC10946217; doi:10.3205/zma001660)
Supplement: Personal life planning depending on position and gender [file JME-41-5-s-001.pdf]

Attachment 1: Personal life planning depending on position and gender

**Position, N (%)**	**Residents (N=466)***		**Specialists (N=653)***		**Senior physicians (N=646)***		**Chief physicians (N=230)***	
**Gender**	**male (N=118)**	**female (N=348)**	**male (N=153)**	**female (N=500)**	**male (N=202)**	**female (N=444)**	**male (N=130)**	**female (N=100)**
**Do you have (a) child(ren)? N (%)**								
Yes	43 (40,9)	151 (45,8)	73 (68,2)	325 (77,6)	**154 (80,2)**	**307 (72,4)**	**83 (76,1)**	**51 (55,4)**
No	62 (59,0)	179 (54,2)	34 (31,8)	94 (22,4)	**38 (19,8)**	**117 (27,6)**	**26 (23,9)**	**41 (44,6)**
**Do you want children? N (% of ‚no children‘)**								
Yes	57 (91,9)	158 (91,3)	22 (66,7)	54 (58,7)	23 (62,2)	68 (59,1)	- #	- #
No	5 (8,1)	15 (8,7)	11 (33,3)	38 (41,3)	14 (37,8)	47 (40,9)		
**“I feel like I have to choose between a career and a child.” N (%)**								
Does not apply / Does rather not apply	19 (30,6)	21 (12,1)	12 (36,4)	19 (20,4)	**20 (54,0)**	42 (27,8)	**18 (69,2)**	19 (46,4)
Does somewhat apply	13 (21,0)	41 (23,6)	6 (18,2)	9 (9,7)	8 (21,6)	29 (25,2)	4 (15,4)	4 (9,8)
More likely to apply / Applies completely	**30 (48,4)**	**112 (64,4)**	15 (45,5)	**65 (69,9)**	9 (24,3)	**54 (47,0)**	8 (22,3)	18 (43,9)
**“I base my child planning on my career steps.” N (%)**								
Does not apply / Does rather not apply	9 (16,1)	6 (7,3)	8 (38,1)	6 (11,1)	8 (34,8)	16 (23,6)	- #	- #
Does somewhat apply	8 (14,3)	35 (22,3)	2 (9,5)	7 (13,0)	5 (21,7)	10 (14,7)		
More likely to apply / Applies completely	**39 (69,7)**	**105 (66,9)**	**11 (52,3)**	**41 (75,9)**	10 (43,5)	**42 (61,8)**		
**“Can you imagine taking a break from your job to take parental leave?” N (% of “no children”)**								
Yes, after completing postgraduate medical training*	20 (37,0)	65 (42,2)	8 (42,1)	33 (62,3)	16 (69,6)	57 (87,7)	- #	- #
Yes, during postgraduate medical training*	32 (59,3)	85 (55,2)	10 (52,6)	14 (26,4)	-	-		
No	2 (3,7)	4 (2,6)	1 (5,26)	6 (11,3)	7 (30,4)	8 (12,3)		
**“If you can imagine taking parental leave, how many months would you like to take?” N (% of “no children”)**								
1-2 months	10 (21,7)	2 (1,6)	3 (23,1)	1 (2,6)	5 (8,47)	54 (30,3)	- #	- #
3-6 months	**23 (50,0)**	35 (28,7)	**7 (53,8)**	8 (21,1)	23 (39,0)	18 (10,1)		
7-12 months	8 (17,4)	**66 (54,1)**	2 (15,4)	**20 (52,6)**	23 (39,0)	32 (18,0)		
> 12 months	5 (10,9)	19 (15,6)	1 (7,7)	9 (23,7)	8 (13,6)	74 (41,6)		
**“Did you interrupt your professional career due to parental leave?” N (% of “children present”)**								
Yes, after completing postgraduate medical training*	-	-	28 (43,1)	114 (35,5)	47 (31,3)	127 (42,2)	16 (20,0)	**32 (64,0)**
Yes, during postgraduate medical training*	**30 (76,9)**	**131 (90,3)**	21 (32,3)	168 (52,3)	-	-	-	-
No	9 (23,1)	14 (9,7)	16 (24,6)	39 (12,1)	**103 (68,7)**	**174 (57,8)**	**64 (80,0)**	18 (36,0)

Attachment 1 to Oberberg S, Enax-Krumova E, Kruppa C, Meyer-Frießem C, Denz R, Funhoff A, Behrens V, Berres D, Fortmeier V, Sträter D, Strotmann J, von Glinski M. *Career and life planning in the context of the postgraduate medical training – current challenges and opportunities*. GMS J Med Educ. 2024;40(1):Doc5. DOI: 10.3205/zma001660

**“How long did you take parental leave?” N (% of “parental leave taken”)**								
1-2 months	**11 (36,7)**	1 (0,8)	**11 (36,7)**	3 (1,4)	**23 (39,0)**	18 (10,1)	**11 (73,3)**	3 (9,4)
3-6 months	**14 (46,7)**	10 (7,5)	**11 (36,7)**	19 (8,9)	**23 (39,0)**	32 (18,0)	2 (13,3)	**8 (25,0)**
7-12 months	4 (13,3)	**54 (40,6)**	6 (20,0)	**102 (47,9)**	8 (13,6)	**74 (41,6)**	2 (13,3)	**15 (46,9)**
> 12 months	1 (3,3)	**68 (51,1)**	2 (6,7)	**89 (41,8)**	5 (8,5)	**54 (30,3)**	0	6 (18,8)
**“I believe that parental leave will reduce my chances of promotion in the long term.” N (% of “not yet taken parental leave”)**								
Strongly disagree / Strongly disagree	12 (22,7)	10 (6,5)	4 (21,0)	3 (5,8)	**82 (44,6)**	88 (20,9)	- #	- #
Somewhat true	7 (13,2)	25 (16,3)	2 (10,5)	3 (5,8)	39 (21,2)	77 (18,7)		
Strongly agree / Agree completely	**34 (64,1)**	**72 (77,1)**	**13 (68,4)**	**46 (88,4)**	63 (34,2)	**248 (60,3)**		
**“Taking parental leave will reduce my chances of promotion in the long term” N (%)**								
Strongly disagree / Strongly disagree	12 (41,4)	23 (17,6)	**12 (60,0)**	34 (20,4)	**27 (58,7)**	39 (30,9)	- #	- #
Somewhat true	5 (17,2)	20 (15,3)	2 (10,0)	29 (17,4)	9 (19,6)	26 (20,6)		
Strongly agree / Agree completely	12 (41,4)	**88 (67,1)**	6 (30,0)	**64 (62,2)**	10 (21,7)	**61 (48,4)**		
**Do you work full-time (≥ 100%) or part-time (<100%)? N (%)**								
Part-time	7 (7,5)	98 (31,5)	13 (14,6)	213 (53,5)	25 (13,8)	167 (41,9)	- #	- #
Full-time	86 (92,5)	213 (68,5)	76 (85,4)	185 (46,5)	**156 (86,2)**	**232 (58,1)**		
**Have you ever worked part-time (<100%) during your career? N (%)**								
Yes	- #	- #	- #	- #	- #	- #	18 (17,1)	**42 (46,7)**
No							87 (82,9)	48 (53,3)
**Domestic/family responsibilities are distributed as follows: N (%)**								
I am a single parent	1 (0,1)	13 (4,3)	2 (2,3)	22 (5,9)	3 (1,7)	31 (8,4)	0	6 (12,0)
I am primarily responsible for domestic/ family responsibilities	5 (5,2)	**111 (36,9)**	6 (6,9)	**190 (50,7)**	9 (5,2)	**131 (35,5)**	0	10 (20,0)
Domestic/family responsibilities are shared equally	**48 (50,0)**	**162 (53,8)**	**48 (55,2)**	**151 (40,3)**	**79 (45,7)**	**173 (46,9)**	21 (25,3)	**25 (50,0)**
My partner does most of the domestic/family work	**42 (48,9)**	15 (4,9)	**31 (35,6)**	12 (3,2)	**82 (47,4)**	34 (9,2)	**62 (74,7)**	**9 (18,0)**

* The numbers and % refer to the (percentage) shares of the answered questions. People who identified themselves as "divers" are not listed as a separate item due to the very small number.
# Not surveyed for corresponding career position due to different priorities or question wording.

Attachment 1 to Oberberg S, Enax-Krumova E, Kruppa C, Meyer-Frießem C, Denz R, Funhoff A, Behrens V, Berres D, Fortmeier V, Sträter D, Strotmann J, von Glinski M. *Career and life planning in the context of the postgraduate medical training – current challenges and opportunities*. GMS J Med Educ. 2024;40(1):Doc5. DOI: 10.3205/zma001660
