# Supplementary material for: Career and life planning in the context of the postgraduate medical training – current challenges and opportunities
Source: GMS J Med Educ. 2024 Feb 15;41(1):Doc5. doi: 10.3205/zma001660 (PMC10946217; doi:10.3205/zma001660)
Supplement: Opinion on work-life balance depending on position and gender [file JME-41-5-s-002.pdf]

Attachment 2: Opinion on work-life balance depending on position and gender

Position, N (%)	Residents (N=468)*		Specialists (N=654)*		Senior physicians (N=646)*		Chief physicians (N=234)*	
**Gender**	**male (N=118)**	**female (N=348)**	**male (N=153)**	**female (N=500)**	**male (N=202)**	**female (N=444)**	**male (N=130)**	**female (N=104)**
**"Do you think career opportunities are different for men and women?"**								
Yes	81 (80,2)	309 (97,8)	79 (79,0)	399 (96,6)	144 (78,3)	391 (95,4)	- #	- #
No	20 (19,8)	7 (2,2)	21 (21,0)	14 (3,4)	40 (21,7)	19 (4,6)		
**"Establishing the Scandinavian model by equalizing the length of parental leave for men and women in Germany would help to equalize career opportunities for men and women."**								
Disagree / Strongly Disagree	8 (10,12)	23 (7,5)	19 (22,4)	39 (9,85)	28 (19,6)	45 (2,7)	- #	- #
Partially agree	10 (12,7)	56 (18,4)	12 (15,8)	91 (23,0)	20 (14,0)	73 (18,9)		
Tend to agree /Strongly Agree	61 (77,2)	226 (74,1)	47 (61,8)	266 (67,1)	95 (66,4)	268 (69,4)		
**"Employees with family responsibilities are often accommodated at the expense of employees without family responsibilities."**								
Disagree / Strongly disagree	7 (7,6)	44 (10,9)	16 (17,9)	74 (18,6)	24 (13,3)	63 (15,8)	- #	- #
Partially agree	23 (24,7)	70 (22,5)	17 (19,1)	100 (25,1)	40 (22,1)	112 (28,1)		
Tend to agree / Strongly agree	63 (67,8)	207 (66,6)	56 (62,9)	224 (56,3)	117 (64,6)	224 (56,1)		
**"What do you think about job sharing models (e.g. 2 people sharing a 100% position)?"**								
Is/sounds like a good concept for all levels of hierarchy	50 (53,8)	199 (64,2)	44 (49,4)	266 (67,2)	98 (54,1)	248 (62,3)	43 (40,6)	42 (46,7)
Is/sounds like a good concept for residents and specialists	20 (21,5)	71 (22,9)	21 (23,6)	83 (21,0)	47 (26,0)	84 (21,1)	36 (34,0)	34 (37,8)
Is/sounds like an unworkable concept	23 (24,7)	40 (12,9)	24 (27,0)	47 (11,9)	36 (19,9)	66 (16,6)	27 (25,5)	14 (15,6)

Attachment 2 to Oberberg S, Enax-Krumova E, Kruppa C, Meyer-Frießem C, Denz R, Funhoff A, Behrens V, Berres D, Fortmeier V, Sträter D, Strotmann J, von Glinski M. *Career and life planning in the context of the postgraduate medical training – current challenges and opportunities*. GMS J Med Educ. 2024;40(1):Doc5. DOI: 10.3205/zma001660

**“I believe that taking parental leave can have a negative impact on the long-term promotion opportunities of colleagues/employees.”**<br>Disagree / Strongly disagree<br>Partially agree / Tend to agree / Strongly agree	- #	- #	- #	- #	72 (39,7)<br>43 (23,8)<br>**66 (36,5)**	66 (16,6)<br>86 (21,7)<br>**245 (61,8)**	**43 (40,0)**<br>22 (21,0)<br>**41 (39,0)**	18 (19,9)<br>20 (22,2)<br>**52 (57,7)**
**“Do you support other colleagues in taking parental leave?”**<br>Yes, for both men and women<br>Yes, for both men and women, but only up to two months<br>No, not for men and women equally	- #	- #	- #	- #	139 (76,8)<br>21 (11,6)<br>21 (11,6)	348 (87,7)<br>21 (5,29)<br>28 (7,05)	**83 (79,0)**<br>14 (13,3)<br>8 (7,62)	**87 (96,7)**<br>2 (2.22)<br>1 (1.11)

* The numbers and % refer to the (percentage) shares of the questions answered in each case.
# Not surveyed for corresponding career position due to different priorities or question wording.
