# Supplementary material for: Career and life planning in the context of the postgraduate medical training – current challenges and opportunities
Source: GMS J Med Educ. 2024 Feb 15;41(1):Doc5. doi: 10.3205/zma001660 (PMC10946217; doi:10.3205/zma001660)
Supplement: Views on work-life balance depending on whether you work in a surgical or conservative specialty [file JME-41-5-s-003.pdf]

## Attachment 3: Views on work-life balance depending on whether you work in a surgical or conservative specialty

| Specialty | Physicians working in surgical disciplines, n=449* | Physicians working in conservative medicine, n=848* |
|---|---|---|
| **Do you have (a) child(ren)? N (%)** | | |
| Yes | 295 (65,7) | 595 (70,9) |
| No | 154 (34,3) | 244 (29,1) |
| **Do you want children? N (% of "no children")** | | |
| Yes | 113 (82,5) | 120 (61,2) |
| No | 24 (17,5) | 76 (38,8) |
| **"I have/had the feeling that I had to choose between a career and a child." N (%)** | | |
| Disagree / Strongly Disagree | 39 (25,6) | 78 (32,6) |
| Partially agree | 35 (23,0) | 45 (18,8) |
| Tend to agree /Strongly Agree | **116 (76,3)** | **117 (48,7)** |
| **"I base my child planning on my career steps." N (%)** | | |
| Disagree / Strongly Disagree | 14 (12,5) | 23 (19,2) |
| Partially agree | 10 (8,93) | 28 (23,3) |
| Tend to agree /Strongly Agree | **88 (78,6)** | **69 (57,5)** |
| **"Can you imagine taking a break from work to take parental leave?" N (% of "no children")** | | |
| Yes, after training* | **48 (60,0)** | 28 (37,3) |
| Yes, during training* | 27 (33,8) | **43 (57,3)** |
| Not at all | 5 (6,25) | 4 (5,33) |
| **"If you can imagine taking parental leave, how many months?" N (% of "no children")** | | |
| 1-2 months | 25 (19,4) | 28 (13,5) |
| 3-6 months | 36 (27,9) | 46 (22,2) |
| 7-12 months | 50 (38,8) | 78 (37,7) |
| > 12 months | 18 (13,9) | 55 (26,6) |
| **"Did you interrupt your work due to parental leave?" N (% of "children present")** | | |
| Yes | 149 (58,9) | 350 (69,9) |
| No | 104 (41,1) | 151 (30,1) |
| **"How long did you take parental leave?" N (% of "parental leave taken")** | | |
| 1-2 months | 6 (6,98) | 15 (7,4) |
| 3-6 months | 18 (20,9) | 18 (8,9) |
| 7-12 months | **35 (40,7)** | 75 (37,1) |
| > 12 months | 27 (31,4) | **94 (46,5)** |
| **"I believe that parental leave will reduce my chances of promotion in the long term." N (% of "not yet taken parental leave")** | | |
| Disagree / Strongly Disagree | 46 (17,4) | 97 (27,1) |
| Partially agree | 41 (15,6) | 73 (20,4) |
| Tend to agree /Strongly Agree | **176 (67,0)** | **188 (52,5)** |
| **"Taking parental leave will reduce my chances of promotion in the long term" N (%)** | | |
| Disagree / Strongly Disagree | 33 (28,4) | 75 (28,5) |
| Partially agree | 20 (17,2) | 50 (19,0) |
| Tend to agree /Strongly Agree | **63 (54,3)** | **138 (52,5)** |

Attachment 3 to Oberberg S, Enax-Krumova E, Kruppa C, Meyer-Frießem C, Denz R, Funhoff A, Behrens V, Berres D, Fortmeier V, Sträter D, Strotmann J, von Glinski M. *Career and life planning in the context of the postgraduate medical training – current challenges and opportunities*. GMS J Med Educ. 2024;40(1):Doc5. DOI: 10.3205/zma001660

| | | |
|---|---|---|
| **"I believe that taking parental leave can have a negative impact on the long-term career development of colleagues/employees." N (%)** | | |
| Disagree / Strongly Disagree | 58 (25,5) | 98 (24,8) |
| Partially agree | 41 (18,1) | 98 (24,8) |
| Tend to agree /Strongly Agree | **128 (56,4)** | **199 (50,3)** |
| **"Do you support other colleagues taking parental leave?" N (%)** | | |
| Yes, for both men and women | 195 (85,9) | 365 (92,4) |
| Yes, for both men and women, but only up to two months | 17 (7,5) | 11 (2,8) |
| No, same for men and women | 15 (6,6) | 19 (4,8) |
| **"Do you support other colleagues/employees who wish to work part-time?" N (%)** | | |
| Yes, for both men and women | **180 (79,3)** | **373 (86,7)** |
| Yes, for both men and women, but only for a limited period of time | 18 (7,9) | 30 (6,9) |
| No, equally for men and women | 29 (12,8) | 27 (6,3) |
| **Do you work full-time (≥ 100%) or part-time (<100%)? N (%)** | | |
| Part-time | 105 (24,5) | 303 (38,4) |
| Full-time | 323 (75,5) | 486 (61,6) |
| **This is how domestic/family responsibilities are distributed. N (%)** | | |
| I am a single parent | 18 (4,5 | 44 (6,8) |
| I do most of the domestic/family responsibilities | 87 (21,9) | **238 (36,6)** |
| Domestic/family responsibilities are shared equally | **198 (49,7)** | **242 (37,2)** |
| My partner does most of the domestic/family responsibilities | 95 (23,9) | 127 (19,5) |
| **"Do you think that career opportunities are different for men and women?" N (%)** | | |
| Yes | 359 (92,3) | 635 (91,5) |
| No | 30 (7,71) | 59 (8,5) |

* The numbers and % refer to the (percentage) shares of the questions answered in each case.
